# Carbon nanotubes and graphene as counter electrodes in dye-sensitized solar cells

**DOI:** 10.1186/s11671-025-04279-7

**Published:** 2025-06-16

**Authors:** Simon Bbumba, Moses Kigozi, Ibrahim Karume, Solomon Yiga, Hussein Kisiki Nsamba, Muhammad Ntale

**Affiliations:** 1 Department of Chemistry, College of Natural Sciences, Makerere University, P.O. Box 7062, Kampala, Uganda; 2 Department of Science, Faculty of Science and Computing, Ndejje University, P.O. Box 7088, Kampala, Uganda; 3 Department of Chemistry, Busitema University, P. O. Box 236, Tororo, Uganda; 4 Department of Chemistry, Faculty of Science, Muni University, P.O. Box 725, Arua, Uganda

**Keywords:** Platinum, Carbon nanotubes, Graphene, Nanosheets, Nanoplatelets, Fullerenes

## Abstract

Addressing the global demand for cost-effective and sustainable energy sources, dye-sensitized solar cells (DSSCs) have emerged as a promising alternative to conventional silicon-based photovoltaics. However, the use of platinum which is a rare and expensive counter electrode (CE) hinders the widespread application of DSSCs, necessitating the use of cheap, abundant, and efficient materials. The review therefore focuses on carbon-based nanomaterials specifically carbon nanotubes (CNTs) and graphene as CEs in DSSCs. The CE plays a vital role in regenerating the redox couple, and its charge transfer resistance (Rct) should ideally be 1 Ω cm² for optimal performance. Carbon nanotubes comprising single-walled carbon nanotubes (SWCNTs), double-walled carbon nanotubes (DWCNTs), and multiwalled carbon nanotubes (MWCNTs) are mainly prepared by chemical vapor deposition (CVD). The SWCNTs have achieved an efficiency of 7.79%, comparable to platinum electrodes, and this was due to the morphology, which influenced the redox mediator regeneration but also reduced the R_ct_. In addition, graphene with high transparency (97.7%), large specific surface area (2630 m^2^ g^− 1^), excellent thermal conductivity (3000 W m^− 1^ K^− 1^), and good carrier mobility properties (10,000 cm^2^ V^− 1^ S^− 1^) have also been applied. In this, the Graphene nanosheets demonstrated a 6.81% efficiency, comparable to platinum (7.59%) due to a high open circuit voltage (V_oc_), which accounts for the reduction of iodide/triiodide redox couple. Lastly, the Graphene nanoplatelets demonstrated a 9.3% efficiency comparable to that of Platinum 7.53% due to low charge transfer resistance, high electrocatalytic activity, and good fill factor.

## Article highlights

- Single-walled carbon nanotubes (SWCNTs) as counter electrodes in dye-sensitized solar cells (DSSCs) have achieved a power conversion efficiency of 7.79%, comparable to traditional platinum electrodes, attributed to their morphology which enhances redox mediator regeneration and reduces charge transfer resistance.
- Graphene nanosheets demonstrated a DSSC efficiency of 6.81%, close to platinum’s 7.59%, primarily due to a high open-circuit voltage resulting from efficient reduction of the iodide/triiodide redox couple.
- Graphene nanoplatelets achieved an impressive 9.3% efficiency in DSSCs, surpassing platinum’s 7.53%, due to their low charge transfer resistance, high electrocatalytic activity, and good fill factor.

## Introduction

The rapid increase in global energy consumption has intensified the demand for sustainable and efficient energy sources [1–3]. The commonly used energy sources are non-renewable, expensive and cause environmental pollution [4]. Among fossil fuels, coal is a major contributor to environmental pollution due to its emission of toxic gases and particulate matter [5]. Hydo electric power, wind, geothermal energy, biomass, and solar radiation are some renewable sources applicable to energy production [6]. Energy from the sun (solar) is readily available, non-toxic, has no emission of greenhouse gases, and is inextinguishable, making it a better alternative [7]. The sun’s solar radiation, which is 3 × 10^4^ J per year, is ten times more than the energy demand needed on Earth [8]. In 1954, Bell Laboratories designed the first photovoltaic device with a p-n junction that had an efficiency of 6% [9, 10]. It is worth menioning that solar radiation faces a few challenges, such as efficiency, storage, and cost of production [11].

Photovoltaic (PV) technology, due to charge separation phenomena within the semiconductor of the cell, can convert solar radiation into electrical energy [12, 13]. The working principle of all photovoltaic devices and semiconductors depends on the Photoelectric Effect [14]. The classification of PV technology is based on three generations: first, second, and third generation [15]. The first-generation PV is made of silicon-based materials comprising either monocrystalline or polycrystalline [16]. Silicon solar cells have a photoconversion efficiency (PCE) of 25%, according to labexperiments, and 22% when it comes to ommercial applications [17]. The second generation comprises non-crystalline cadmium telluride (CdTe), copper gallium indium diselenide (CIGS), and amorphous silicon. The theoretical value of first- and second-generation solar cell photo conversion efficiency is 33% based on the SchokleyQueisser limit [18]. Compared to the first generation, these have low manufacturing costs and a large surface area for deposition. The third generation, which was developed to solve the disadvantages of the first and second-generation cells, comprises quantum dots (QDs) [19, 20], dye-sensitized solar cells (DSSCs) [12, 21], perovskite solar cells (PSCs) [22] and bulk heterojunction solar cells (BHJ). Third-generation solar cells’ advantages are low cost, abundance, and simplicity in design during the fabrication of flexible and non-flexible solar cells [14]. In DSSCs, dye molecules are either organic or inorganic-organic, QDs whose bandgap is tunable employ inorganic quantum dots as sensitizers. Furthermore, the BHJ has conductive polymers that play the role of donor while the large carbon materials are acceptors [23]. From this analysis, the DSSCs are gaining more attention for commercialization since they are printable on both flexible and rigid substrate surfaces.

**Fig. 1:**
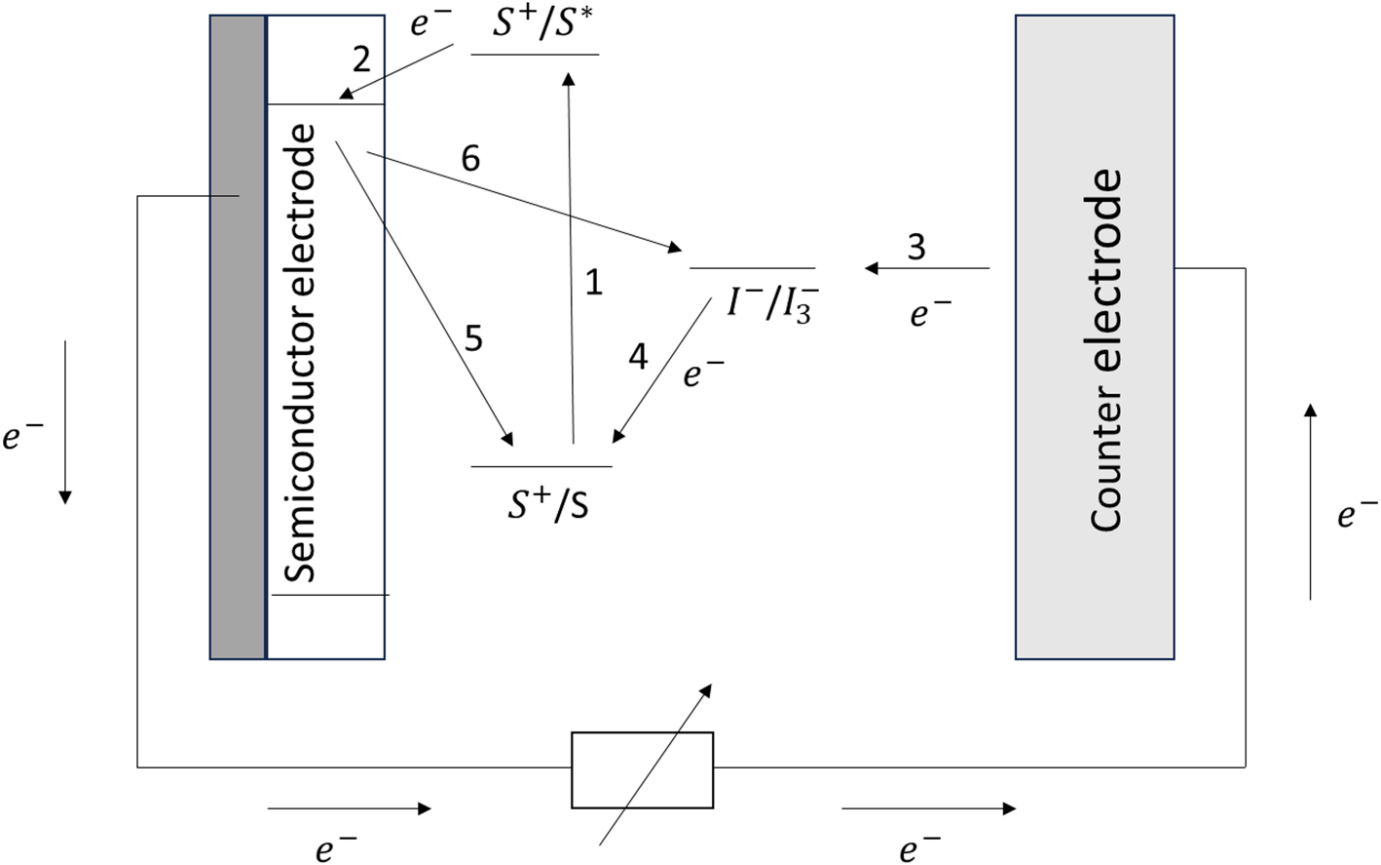
The schematic working principle of DSSC

Upon the introduction of the third generation PV devices by O’Regan and Grätzel in 1991, they found wide application due to the ability to achieve an efficiency of almost 10% [24], low production and fabrication cost [25]. The DSSCs comprise five significant components, as shown in Fig. 1, i.e., an electrolyte [26], visible light adsorber dye [27], counter electrode [28], transparent conductive oxide substrate [29], and nanostructured semiconductor (n-type) [30]. When exposed to solar energy, the dye absorbs a photon causing an electron to become excited and migrate from the highest to the lowest unoccupied molecular orbital of the dye molecule [31]. It is followed by injection into the band of the photoanode which is then moved to the transparent conductive oxide through which it is transported to the CE via the external circuit [32]. The electrolyte then gives an electron to the sensitizer as compensation for its electron migration to the semiconductor’s conduction band. This then causes the electrolyte to regenerate and return to its normal state and the cycle continues [33].

During the fabrication process of the DSSCs, the CE is an essential component since it returns the electron into the cell, which reduces the electrolyte redox couple (triiodide to iodide). It has been observed that platinum electrodes have relatively high efficiency [34]. However, the high price and unavailability of platinum have hindered its use as a CE; hence, other alternative materials have been applied to replace platinum as the CE [35–37]. Alternative sources include conductive polymers, carbonaceous material, and metal oxides, all of which have been applied in DSSCs to replace platinum [38]. The carbonaceous materials include carbon nanotubes [39], carbon quantum dots [40], carbon black [41], graphene [42], carbon nanofibers [43], carbon nano-onions [44], carbon nano-horns [45] and amorphous carbon [46]. This review aims to illustrate the use of CNTs and graphene as CEs in the design of DSSCs.

## Working principle and characterization of DSSCs

The DSSCs contain five components: the transparent conductive oxide, which acts as a support; the semiconductor, usually TiO_2_, the dye sensitizer molecule, which absorbs a photon of light; In addition, the electrolyte solution, having a mediator and carries out regeneration of the dye and the CE commonly platinum regenerates the redox mediator. Figure 1 shows the working principle of the DSSC.

The sensitizer (S) absorbs a photon of energy (Eq. 1) that causes the injection of an electron into the semiconductor conduction but also causes oxidation (Eq. 2).

1 $$\:{S}_{\left(adsorbed\right)}+h\nu\:\to\:\:{S}_{\left(adsorbed\right)}^{*}$$

2 $$\:{S}_{\left(adsorbed\right)}^{*}\to\:\:{S}_{\left(adsorbed\right)}^{+}+\:{e}_{\left(injected\right)}^{-}$$

It is observed that the electron flows through the external circuit via the load to reach the CE, which reduces the redox mediator (Eq. 3), followed by the regeneration of the sensitizer into its ground state (Eq. 4).

3 $$I_{3}^{ - } + 2\cdot~e_{{\left( {cathode} \right)}}^{ - } \to ~3I_{{\left( {cathode} \right)}}^{ - }$$

4 $$S_{{\left( {adsorbed} \right)}} + \frac{3}{2}I^{ - } \to ~S_{{\left( {adsorbed} \right)}} + ~\frac{1}{2}I_{3}^{ - }$$

The output of the DSSC is affected by the recombination of the injected electron by the oxidized dye molecule (Eq. 5) or the oxidized redox mediator (Eq. 6).

5 $$S_{{\left( {adsorbed} \right)}}^{ + } + e_{{\left( {TiO_{2} } \right)}}^{ - } \to ~S_{{adsorbed}}$$

6 $$I_{3}^{ - } + 2\cdot~e_{{\left( {TiO_{2} } \right)}}^{ - } \to ~3I_{{\left( {anode} \right)}}^{ - }$$

Recombination affects efficiency when an excited electron returns to the electrolyte solution without reaching the photoanode or gets trapped in the defect states, thus resulting in low conversions [47].

**Fig. 2:**
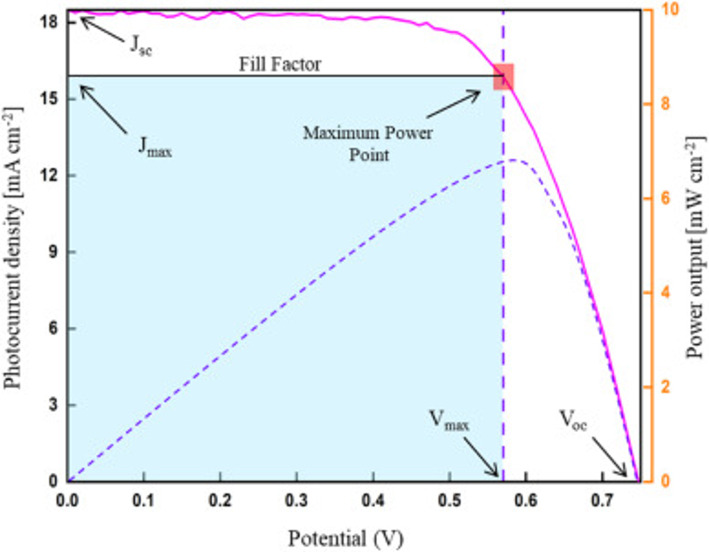
A diagram of the photocurrent density-voltage curve. Adapted with permission ref [49]. Copyright 2024, Elsevier

It is observed that the DSSC working principle and performance are influenced by cost and conversion efficiency. It is worth noting that several techniques govern the working principle of the DSSCs. Fill factor (FF), short circuit current density (J_sc_), theoretical power (PT), open circuit voltage (V_oc_), and maximum power point (Pmax) [48]. A photocurrent density-voltage (J-V) curve is used by exposing the DSSC to a standard air mass (AM). From the J-V curve, the current density at maximum (J_max_), V_oc_, J_sc_, maximum power point (P_max_), and the voltage at maximum power point (V_max_) are obtained as shown in Fig. 2 [49].

V_oc_ is the maximum voltage and is expressed through the difference between an open circuit condition and the electrodes of the solar cell. At open circuit conditions in the presence of light, there’s variation in the two terminals, which gives rise to the open circuit voltage, as shown in (Eq. 7).

7 $$\:\text{V}\text{o}\text{c}=\frac{E}{e}+\frac{{K}_{B}\:T}{e}\:.\:In\:\left(\frac{n}{{N}_{CB}}\right)-\:\frac{{E}_{redox}}{e}$$

Where E_redox_ is the redox potential of the redox mediator, is the number of electrons in the conduction band, k_B_ is the Boltzmann constant, e is the elementary charge of the electrons, T is the absolute temperature, and N_CB_ is the density of accessible states at the conduction band [50].

Hence, the maximum V_oc_ of a DSSC corresponds to the difference between the energy level (E_CB_) of the photoelectrode’s Fermi level and the electrolyte’s redox potential (E_redox_). At absolute temperature, the collection of energy levels is described by the Fermi level. The actual V_oc_ is observed to be lower than the theoretical during dye recombination [50]. At similar temperature and light conditions, the open circuit voltage is constant for any solar cells. A maximum J_sc_ is achieved when the cell is short-circuited, whereas the open-circuit voltage is obtained in an open circuit where no current flows [51].

Furthermore, the J_sc,_ which is the short circuit current density, has units of milliamperes per square centimetre (mA/cm^2^) and is obtained on light radiation in a short circuit. Jsc is the maximum current obtained, and the electron movement is not opposed by the voltage. The J_sc_ is influenced by the absorption coefficient of the sensitizer, photoanode particle, and dye sensitizer molecule. The molecular structure and electrochemical properties of the dye sensitizer greatly influence the J_sc_. The surface functional groups of the sensitizer create a strong bond with the photoanode which leads to a high light energy absorption which in turn increases the short circuit voltage. In addition, regulating the molecular location and dye concentration (composition) also increases the J_sc_.

In addition, the solar cell’s overall quality is determined by a parameter known as the fill factor (FF) [52]. and is described by (Eq. 8).

8 $$\:\text{F}\text{F}=\frac{{\text{J}}_{\text{m}}\times\:\:{\text{V}}_{\text{m}}}{{\text{J}}_{\text{S}\text{C}}\:\times\:{\:\text{V}}_{\text{O}\text{C}}}$$

The operational losses due to electrochemical and electrical processes are reflected by the FF, where V_m_ and J_m_ are the maximum voltage and current, respectively. An ideal diode has a unity fill factor. The FF in DSSC generally lies between 0.6 and 0.75 due to transport losses and charge recombination [53]. Improving the shunt resistance and reducing the series resistance is essential to achieve a better fill factor. This results in better charge transfer and diffusion, which leads to a reduction in the overvoltage [54].

Another critical parameter is the energy conversion efficiency (ECE), which is obtained when the incident power of the cell is split at a specific point [55] and denoted by η. This parameter is used to understand, compare and evaluate the performance of different solar cells. It highlights the information about electricity generation through the conversion of solar radiation and photovoltaic parameters. The ECE of a solar cell is mainly determined by the temperature, distribution of the spectral intensity and overall light during illumination conditions [56]. It is observed that when these factors fluctuate, they result in a change in the ECE. In the last twenty years, there has been no major advancement to the ECE, and regarding reports from the Renewable Energy Laboratory (NREL), the best efficiency is about η = 11.9% [57].

The power conversion efficiency (PCE) is the ratio of the output of the solar cell’s highest energy to the input of the energy from solar radiation. The maximum amount of power a solar cell can produce is equal to the product of the maximum current and maximum voltage (Eq. 9).

9 $$\:{P}_{max}={J}_{m}\times\:\:{V}_{m}$$

PCE under sunlight irradiation is obtained using (Eq. 10)

10 $$\:{\upeta\:}=\frac{{P}_{max}}{{P}_{in}}=\:\frac{{J}_{m}\:\times\:{\:V}_{in}}{{P}_{in}}=\:\frac{{J}_{SC}\:\times\:{V}_{OC}\:\times\:FF}{{P}_{in}}$$

Where Pin is the power per unit area of the incident light.

The operation temperature, intensity, and incident light spectrum greatly influence the performance of the solar cell [58].

Perhaps another critical parameter is the IPCE, which determines the percentage of conversion of absorbed light to current. It gives information about the number of electrons moving through the external circuit under short circuit conditions per incident light (Eq. 11) [59, 60].

11 $$\:\text{I}\text{P}\text{C}\text{E}\:=\:1240\:\left(\text{V}\text{*}\text{n}\text{m}\right)\:\times\:=\frac{{J}_{sc}\:\left(\lambda\:\right)\:[A/{cm}^{2}]}{{\lambda\:\:\left[nm\right]\times\:P}_{in\:[W/{cm}^{2}]}}$$

Where $\:\lambda\:$ is the wavelength, J_sc_ is the short-circuit current density, and Pin is the power per unit area of the incident light. It is worth noting that factors such as irradiation temperature, illumination intensity, and humidity influence photovoltaic performance.

## Counter electrodes in DSSCs

It is a crucial part of the DSSC since it collects electrons from the external circuit, reduces triiodide to iodine, and regenerates the dye sensitizer. In some materials, sunlight is also utilized by reflecting it into the cell to excite an electron from the sensitizer. The CEs in DSSCs have been prepared using in-situ polymerization, which is normally used for conductive polymers and occurs when an initiator is added to a reaction mixture containing organic monomer and conductive glass substrate to have in-situ growth on the substrate surface. Another method is thermal decomposition, in which the precursor is deposited onto the fluorine-doped tin-oxide and then subjected to high temperatures in a furnace, which triggers decomposition, creating particles that act as CEs. In addition, chemical reduction in which a metal precursor is added to a reducing agent such as hydrazine leads to the formation of metallic nanoparticles, which are then deposited on the current collector. Another method is sputter deposition, which is a physical vapor deposition method where a target material from the source is ejected onto the substrate through sputtering.

Furthermore, chemical vapor deposition is when several volatile precursors decompose on the substrate surface to form the CE. Hydrothermal reaction is when an aqueous medium reacts at high temperature and pressure. Lastly, electrochemical deposition occurs primarily in an electrochemical cell where a metal precursor in the electrolyte solution undergoes reduction due to the application of a current causing deposition on FTO, leading to the formation of the CE. The method of preparation of the counter determines its catalytic and conductivity behaviour as these are influenced by the size, morphology, and surface area, and the reduction of the electrolyte plus efficiency are the outcomes. The larger the surface area and smaller the particle sizes of the CE, there is an observed improved cell efficiency due to the increased active sites [61].

The catalytic activity determines the rapid reaction, reducing the overpotential, which is responsible for the charge transfer resistance R_ct,_ which occurs at a specific current density. For a more efficient CE, the charge transfer resistance should be 1 Ω cm^2^ [62]. The most applied CE is platinum; however, its rarity and high costs have prompted the use of other materials [63]. Alternative materials, such as conducting polymer membranes, carbonaceous materials, sulfides, and metal oxides, are being applied as CEs, although their efficiency is low compared to Platinum [36, 64].

### Platinum counter electrode

Platinum, a rare and expensive noble metal, has been applied as a catalytic component because of its durability and high electrolytic activity, which reduces triiodide to iodide [65]. Several alternative techniques have been applied in the deposition of platinum onto the substrate, including electrodeposition [66, 67], sputtering [68, 69], thermal deposition, and chemical reduction [70, 71]. The preparation methods have a high influence on the morphology, particle size, surface area, and catalytic properties of the electrodes. Table 1 shows how a number of synthesis techniques affect the performance parameters of DSSCs.

**Table 1:** Preparation techniques and their influence on the photochemical parameters

Technique	J_sc_ (mAcm^− 2^)	V_oc_ (V)	FF (%)	η (%)	Reference
Thermal decomposition	15.1	0.760	70.8	8.15	[72]
Electrochemical deposition	16.78	0.660	66.0	7.32	[73]
Chemical reduction	15.22	0.760	73.5	8.51	[74]
Hydrothermal reaction	17.89	0.760	68.0	9.24	[75]

All the preparation techniques have been found to be widely applicable because the conversion efficiency is high for all. Methods are chosen based on the availability and need for a given experiment [76]. In addition, compared to all the synthesis techniques, hydrothermal is the most commonly applied due to its simplicity in preparation, ease of scalability, and low economic value [77]. This method has further been used to synthesize CEs with different sizes, morphology, and sizes such as (nanorods, nanotubes, nanospheres, and nanofibers) [78].

Table 2 shows a comparative study of the overall performance of DSSCs containing platinum, carbon nanotubes and graphene CEs.

**Table 2:** Overall performance of DSSCs devices containing different CEs

Counter electrode	Redox couple	Sensitizer	J_sc_ (mAcm^− 2^)	V_oc_ (V)	FF (%)	η (%)	Reference
Platinum	$\:{I}^{-}/{I}_{3}$	N719	15.20	0.728	69.9	7.70	[79]
Carbon nanotubes	$\:{I}^{-}/{I}_{3}$	N719	11.84	0.682	59.9	4.83	[79]
Graphene	$\:{I}^{-}/{I}_{3}$	N719	12.2	0.640	43.0	4.17	[80]

The DSSC with platinum electrodes has the highest performance compared to graphene and carbon nanotubes, which is attributed to its transparent nature, which leads to a decrease in internal resistance and, hence, high conversion efficiency [79]. Furthermore, platinum has demonstrated a high stability with the traditional redox shuttle (I^−^/I_3_) but also no decrease in efficiency is observed during simulation conditions [81, 82]. It is worth noting that platinum is preferred due to its high electrocatalytic activity for (I^−^/I_3_) and superior conductivity [34].

### Carbon

**Fig. 3:**
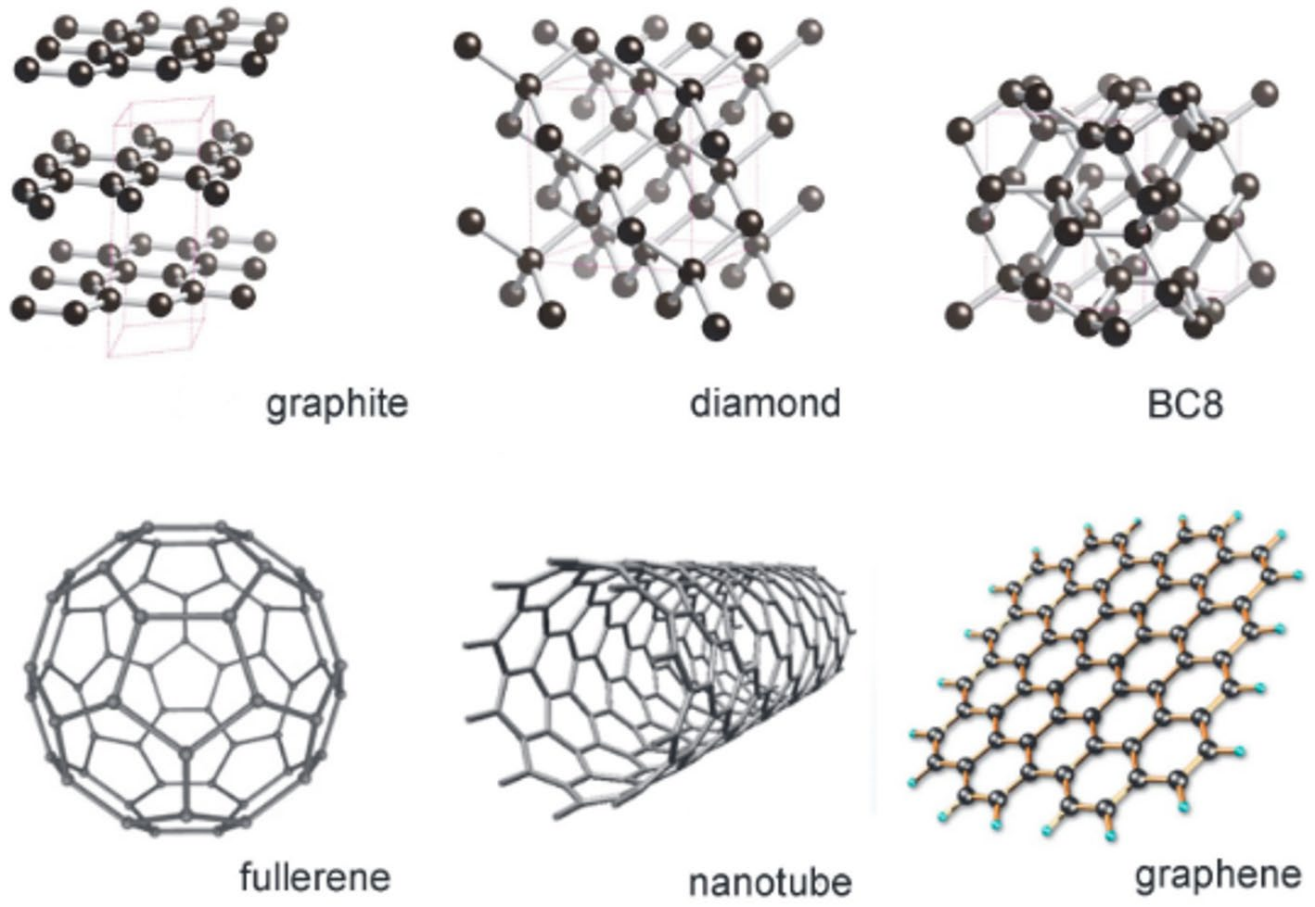
Structures of various allotropes of carbon. Adapted with permission ref [83]. Copyright 2018, De Gruyter

It is one of Earth’s most abundant elements, and it forms bonds with different elements in sp, sp^2^, and sp^3^ hybrid orbitals, giving rise to many stable structures. It has several allotropes, which include graphite, diamond, BC8 (super diamond), fullerene, carbon nanotubes, and graphene, as shown in Fig. 3 [83].

Carbon CEs are cheap, readily available, have a large surface area, have good catalytic activity, are highly corrosion stable, and have good thermal capabilities [84]. Single-wall carbon nanotubes, multiwall carbon nanotubes, graphene, carbon dots, activated carbon, carbon horns, and graphite have been used as CEs. These allotropes of carbon come in several dimensions, including 0D, 1D, 2D, and 3D. The 0D is mainly composed of fullerenes with simple synthesis techniques [85]. The carbon nanotubes, wires, rods, and fibres are under 1D, and these have the most significant application during the fabrication of DSSCs. In addition, the 2D, which has a more complicated fabrication process than the 0D/1D, comprises the flakes and sheet films of graphene, and the 3D consists of graphite molecules. The properties of the different allotropes of carbon are shown in Table 3.

**Table 3:** The different allotropes of carbon and their properties

Allotrope	Hybridization	Young’s modulus	Electron mobility (cm^2^/Vs)
Graphene	sp^2^	1.06	200,000
Fullerene	sp^2^	53–69	0.4-1
Carbon nanotubes	sp^2^	1.033–1.042	1,20,000
Diamond	sp^3^	1220	1800
Graphite	sp^2^	0.795	1.5 * 104

These properties enable carbon to be applied as a low-cost CE since a high electron mobility and low Young’s modulus lead to a reduction in the recombination effects and good charge carrier mobility, which improves the overall cell efficiency [86]. These properties are observed in graphene and carbon nanotubes, which are excellent CE materials [87].

Carbon atoms in sp^2^ hybridization form planar, trigonal structures, creating a delocalized π-electron system that facilitates electron transport and interaction with the redox species but also influences the porosity and surface area of the CE [63]. While sp3 hybridized carbon atoms lack the delocalized π-electron system, resulting in lower conductivity [88].

**Fig. 4:**
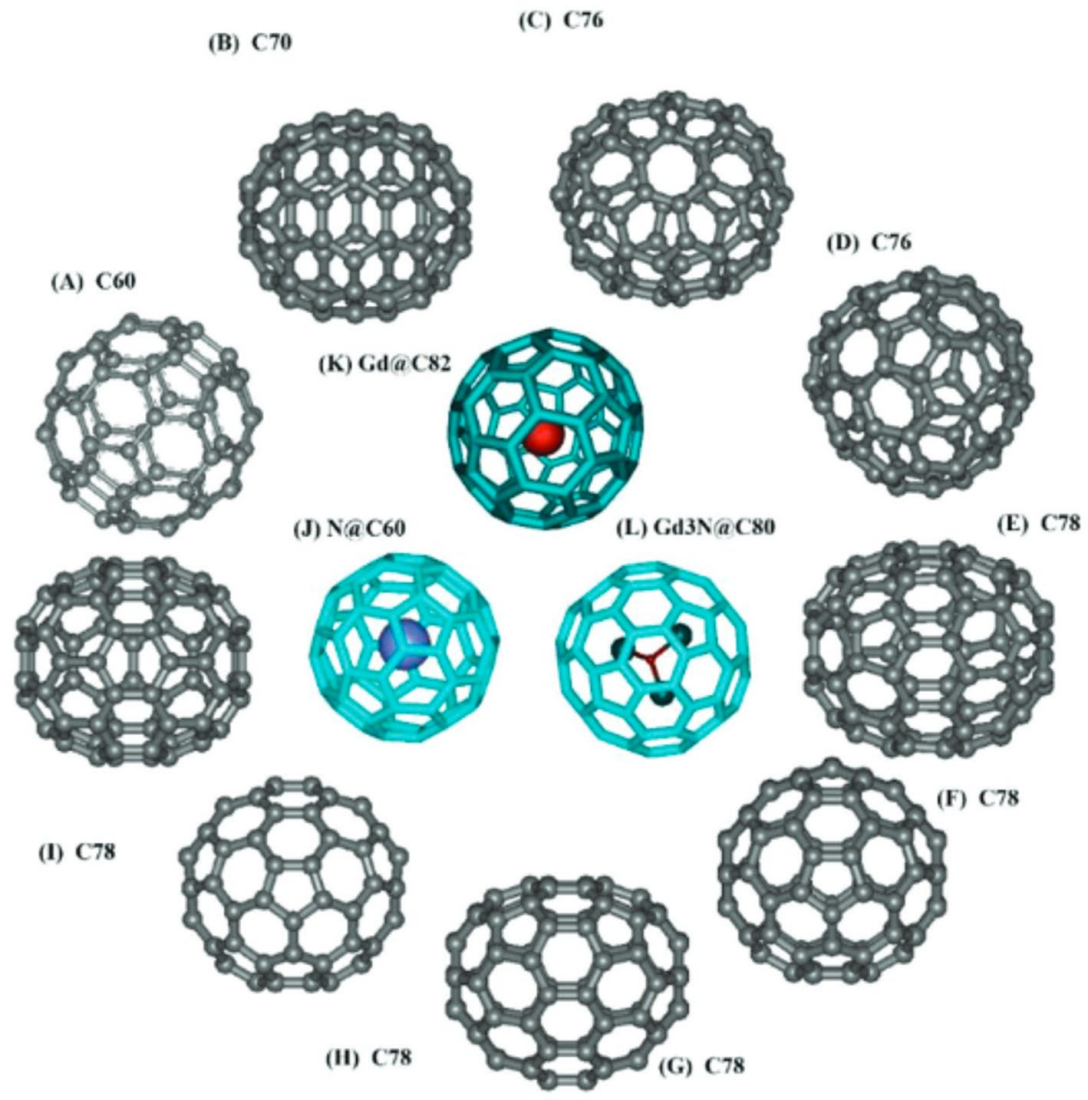
The different fullerene structural models. Adapted with permission ref [91]. Copyright 2006, American Chemical Society

The zero-dimension (0D) carbon forms the fullerenes, which are large molecules consisting of varying numbers of carbon atoms that, through bonding, have an icosahedron symmetry that accounts for the sphere-like structure [89]. C_60,_ whose sphere diameter is 0.7 nm, has 20 hexagons with C5-C6 double bonds and 12 pentagons with C5-C5 single bonds, in which sp^2^ hybridizations influence the bonding [90]. Fullerenes consist of allotropes with more carbon atoms, including C_70,_ C_76,_ C_78,_ C_80,_ and C_82,_ as shown in Fig. 4 [91]. These have been known to have encapsulation properties due to their hollow nature; hence, they are classified as endohedral fullerenes [89].

**Fig. 5:**
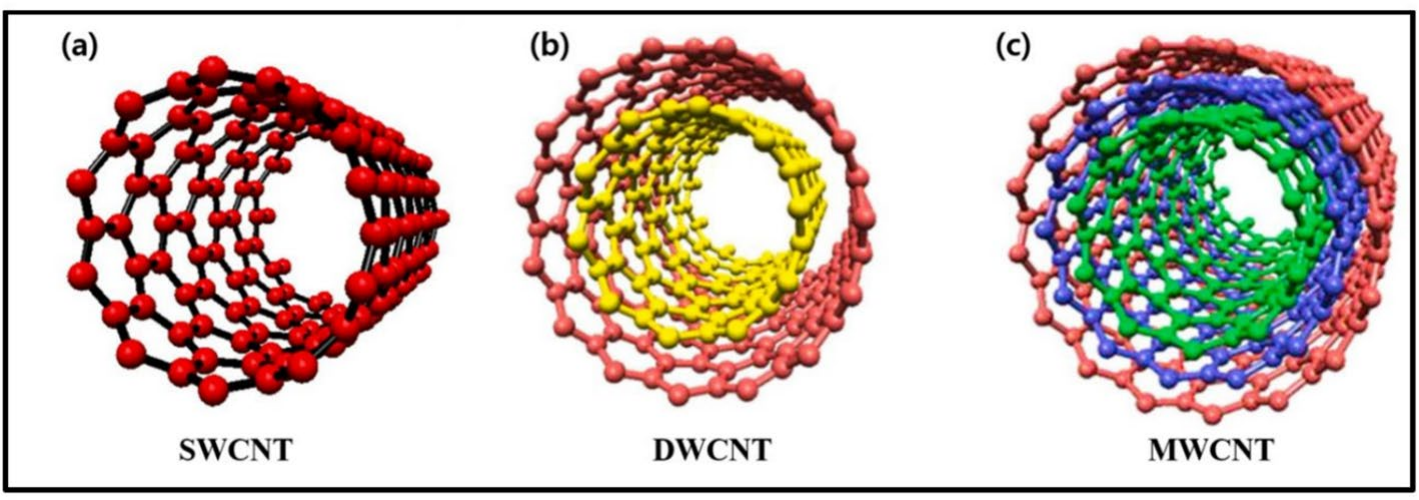
Structure of single-walled carbon nanotubes (**a**), double-walled carbon nanotubes (**b**), and multiwalled carbon nanotubes (**c**) [95]

In addition, the one-dimension carbon allotropes are made up of carbon nanotubes (CNTs), described as graphite sheets rolled into cylindrical shapes. They comprise 60 carbon atoms, which are thus derivatives of fullerenes and carbon fibres that give rise to muffled tubes [92]. CNTs are cylindrical hollow structures obtained when moving single or multilayer graphene sheets [93, 94]. It has forms such as single-walled carbon nanotubes (SWCNTs), multiwalled carbon nanotubes (MWCNTs), and double-walled carbon nanotubes (DWCNTs) as shown in Fig. 5 [95].

For the application of these carbon nanotubes as CEs in DSSCs a number of synthesis techniques are used. Synthesis of CNTs involves three methods, as shown in Table 4 that is chemical vapor deposition (CVD), which requires hydrocarbon substrates such as methane and ethylene to be put in an oven at temperature ranges of 700–900 ^o^C, Arc discharge operates at high temperature > 3000 °C which forms a plasma by evaporation of the carbon atom leading to the formation of SWCNTs and MWCNTs and laser ablation which employs graphite at temperatures of 1200 ^o^C in an electric furnace.

**Table 4:** Comparison of the three most common carbon nanotube synthesis techniques [96]

Method	Temperature (°C)	Diameter (nm)	Purity	Energy	Advantages	Disadvantages
CVD	700–900	20–25	High	Moderate	Large scale production	Defects in MWCNTs
Arc Discharge	1700	4–30	High	High	High quality	Tangled nanotubes
Laser Ablation	1200	10–20	High	High	High purity	Limitation of upscaling

**Fig. 6:**
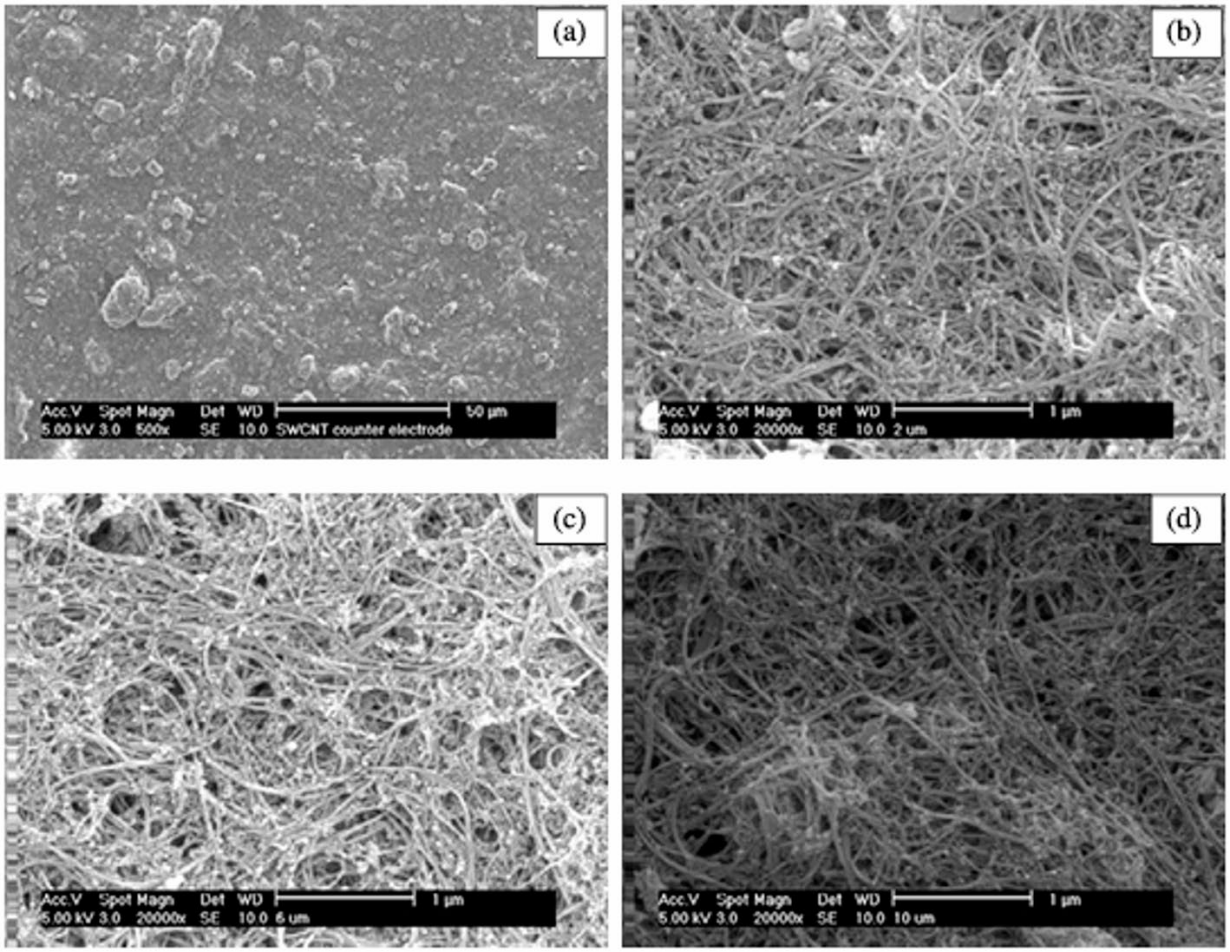
SEM image showing different thickness of SWCNTs, **a**, **b** 2 μm, **c** 6 μm, and **d** 10 μm. Adapted with permission ref [100]. Copyright 2010, IOP Publishing

Carbon nanotubes have good mechanical strength, electrical stability, and conductivity and thus have found wide applications in optics, nanotechnology, electronics, and material science [97]. For the first time Suzuki et al. [98] designed a SWCNTs CE for DSSCs, which had an efficiency of 4.5%, comparble to platinum-sputtered CE with similar conditions. Lee et al. [99] synthesized MWCNTs with low charge transfer resistance and good FF due to good electron mobility; thus, an efficiency of 7.7% was achieved Mei et al. [100] synthesized SWCNTs and MWCNTs by mixing them with polyethylene glycol (PEG) and then put in a mortar, sonicated, and centrifuged. The paste obtained was doctor bladed onto FTO substrate, and PEG was removed through heating at 250 °C for 10 min and 450 °C for 30 min to obtain 2 μm, 6 μm, and 10 μm SWCNTs as shown by the scanning electron microscopy image in Fig. 6.

The SWCNTs morphology did not change as observed with different thickness values; however, bundles are observed at high magnification with a generally rough surface. This promotes easy penetration of the electrolyte solution, improving the electrical conductivity and the regeneration of the redox mediator [100]. Furthermore, the bundles create a highly porous structure for easy diffusion. Still, SWCNTs’ small diameter and hydrophobic nature also induce capillary action, drawing electrolytes into the pores and facilitating their transport.

The thickness of the SWCNTs influences the overall efficiency of the DSSCs, as shown in Table 5.

**Table 5:** SWCNTs thickness effect on the photochemical parameters [100]

Thickness (µm)	V_oc_ (V)	J_sc_ (mAcm^− 2^)	FF (%)	η (%)
2	0.724	14.13	70.0	7.15
6	0.735	14.39	73.0	7.71
10	0.733	14.42	74.0	7.79

In comparison, the SWCNTs have a large surface area, hence low charge transfer resistance compared to the MWCTs, which arises due to the different photochemical parameters as shown in Table 5. It observed that V_oc_ and J_sc_ increase as the thickness increases.

Ahmad et al., [101] prepared carbon nanotubes as CEs and obtained an efficiency of 8.51%, V_oc_ (0.760 V), FF (73%), nd J_sc_ (15.36 mA/cm^2^) which values gave an efficiency which is slightly higher than that of platinum (7.95%). he high efficiency is attributed to the good photochemical parameters of the counter electrode and low transfer resistance.

Another group by Hasan et al., [102] synthesized MWCNTs as CEs for DSSCs and obtained an efficiency of 4.25% whch is comparable to that of platinum (5.53%) wich is mainly brought about by the large surface area.

In addition, graphene is a flat monolayer with sp^2^ hybridized carbon atoms tightly packed in a honeycomb lattice, which is the building block for most allotropes of carbon, i.e., fullerenes, carbon nanotubes, charcoal, and graphite [103]. Due to its unique nature of high transparency (97.7%) [104], high specific surface area (2630 m^2^ g^− 1^) [105], excellent thermal conductivity (3000 W m^− 1^ K^− 1^) [106], Young’s modulus (1 TPa) [107] and good carrier mobility properties (10,000 cm^2^ V^− 1^ S^− 1^) [86]. Because of its high surface area, graphene can reduce the charge transfer resistance and is one of the most promising candidates for replacing platinum [28].

The preparation of graphene includes techniques such as graphite oxide thermal exfoliation [108, 109], electrophoretic deposition followed by annealing [110–113], oxidative exfoliation [114, 115], graphene oxide colloids chemical reduction in microwave-assisted irradiation [116–118]. Table 6 shows the different photochemical parameters and how they vary with the various preparation techniques.

**Table 6:** Graphene Preparation techniques and the influence on the photochemical parameters

Technique	*R*_ct_	J_sc_ (mAcm^− 2^)	V_oc_ (V)	FF (%)	η (%)
Electrophoretic deposition	38	14.30	0.54	65.3	5.69
Oxidative exfoliation	1.20	16.99	0.75	53.6	6.81
Thermal exfoliation	11.7	7.70	0.68	54.0	2.82
Chemical reduction	0	6.12	0.64	56.0	2.19

In comparison from Table 6, it is observed that the best preparation technique for graphene CEs is oxidative exfoliation of graphite followed by hydrazine reduction, which gives an efficiency of 6.81% Zhang et al. [119]. From his work on graphene, a low R_ct_ (1.20) and low diffusion resistance due to decreased internal resistance caused an increase in the Fill factor (53.6). It is observed that the DSSC performance of graphene CE is determined by the physical properties of the fabricated material, i.e. (nanosheets, nanoplatelets, nanofoam, quantum dots, ribbons, aerogels), redox couple and dye sensitizer [120] as shown in Table 7.

**Table 7:** Physical forms of graphene and their influence on the efficiency of the DSSCs

Counter electrode	Redox couple	Sensitizer	J_sc_(mAcm^− 2^)	V_oc_(V)	FF (%)	η (%)	Reference
Nano sheets	$\:{I}^{-}/{I}_{3}$	N719	12.66	0.752	57.2	5.45	[121]
Nanoplatelets	$\:{Co}^{2+/3+}{\left(L\right)}_{2}$	Y123	12.70	1.030	70.0	9.3	[122]
Nanofoam	$\:{I}^{-}/{I}_{3}$	N719	12.20	0.710	60.0	5.2	[123]
Quantum dots	$\:{I}^{-}/{I}_{3}$	N3	5.58	0.583	66.0	2.15	[124]
Ribbons	$\:{I}^{-}/{I}_{3}$	N719	11.85	0.780	49.9	4.61	[125]
Aerogels	–	–	11.88	0.730	71.0	6.20	[126]

The diffusion coefficient of the redox couple determines how quickly it can reach the graphene surface and participate in the electron transfer reaction, thus increasing both the V_oc_ and J_sc_ [127]. In addition, the sensitizer must have several anchoring groups to improve electron injection, and the excited state should easily donate an electron to the semiconductor.

**Fig. 7:**
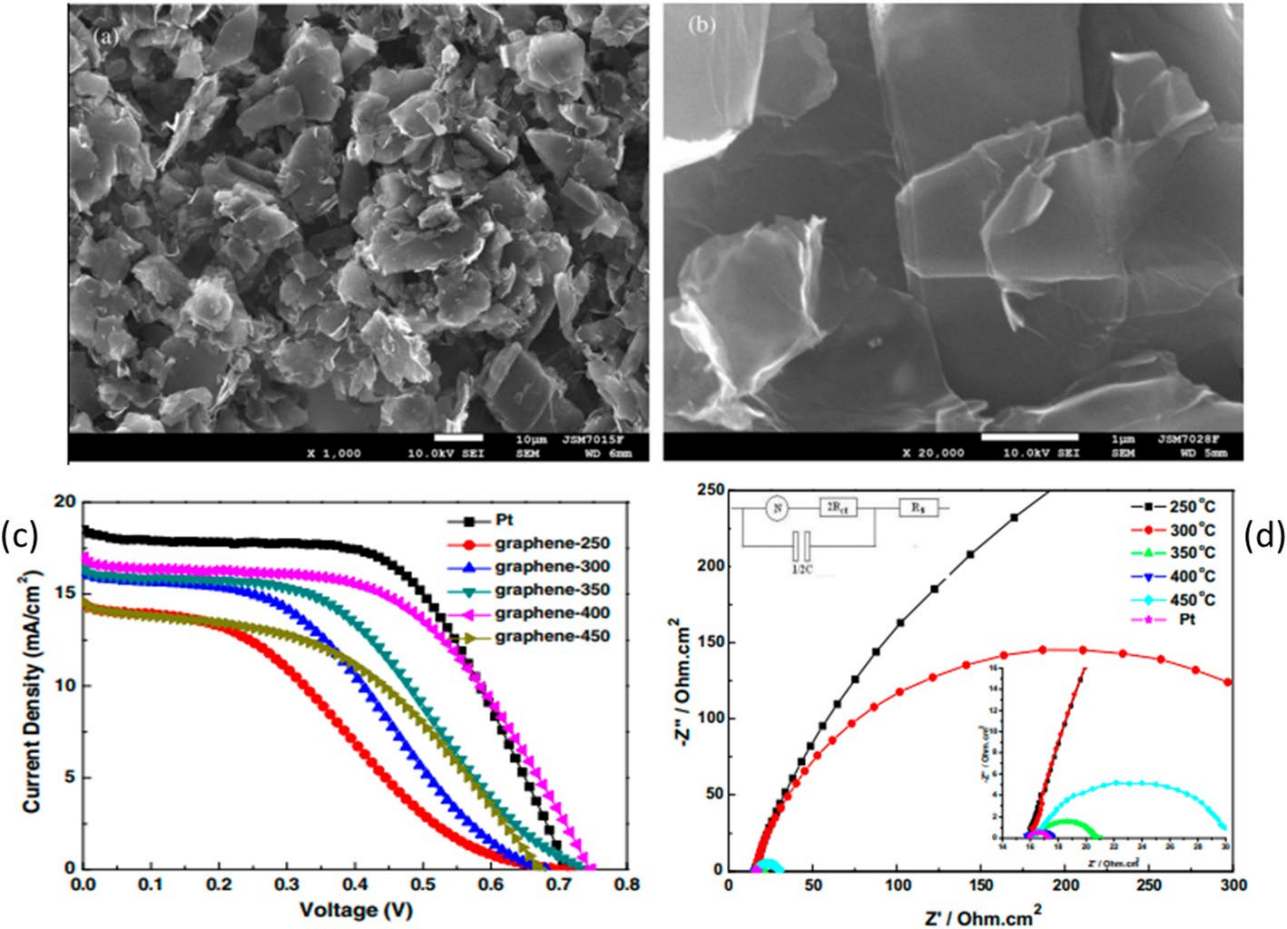
FESEM images of the graphene nanosheets show the morphology, photochemical parameters, and electrochemical characteristics. Adapted with permission ref [119]. Copyright 2011, Elsevier

The presence of structural defects and oxygen-containing sites improves the catalytic activity of the graphene CE [128]. In previous studies, Zhang et al. [119] synthesized graphene nanosheets using a modified Hummer method with an annealing temperature range of 250–450 °C and applied them as CEs in DSSCs. Figure 7 shows the Field emission scanning electron microscopy (FESEM) images, photochemical parameters, and electrochemical characteristics of the graphene nanosheets at temperature ranges of 250–450 °C [119]. Figures 7a and b show the Field emission scanning electron microscopy (FESEM) images of the nanosheets form a three-dimensional structure due to the close packing that accounts for the perpendicular stacking in the nanosheets. This dimensional structure, parallel to the substrate, ensures good adhesion of the nanosheets, but the perpendicularly aligned stacks also quickly reduce triiodide, thus improving the over-performance [119].

Figure 7c shows the photochemical parameters of the graphene nanosheets at different annealing temperatures compared with the platinum CE. The increase in temperature from 250 to 450 °C has a positive effect on the DSSC performance as it improves the J_sc_, V_oc_, FF, and efficiency, as shown in Table 6. The CEs at 400 °C had the highest efficiency comparable with that of platinum because the former has higher V_oc,_ which accounts for the reduction of iodide/triiodide redox couple since it causes a positive shift in the redox energy level [129, 130]. However, at 500 °C, the efficiency decreases because the organic binders between the nanosheets and FTO are burnt to carbon; hence, they easily peel off from the surface. Furthermore, Figure d, which shows the nysquit plot, better explains the variation in I-V curve performance between platinum and graphene at different annealing temperatures. A low charge transfer resistance R_ct_ corresponds to high efficiency and vice versa, causing a decrease. As the annealing temperature increases, the R_ct_ decreases due to a reduction in the number of organic binders on the substrate, as shown in Table 8. At 500 °C, the R_ct_ increases due to poor adhesion onto the substrate, which affects its efficiency.

**Table 8:** Influence of charge transfer resistance on graphene nanosheets and platinum electrode performance [119]

Electrode	*R*_ct_ (Ωcm^2^)	J_sc_ (mAcm^− 2^)	V_oc_ (V)	FF (%)	η (%)
Graphene Nanosheets-250	3337.18	14.478	0.697	32.56	3.29
GrapheneNanosheets-300	274.19	16.147	0.680	40.43	4.44
Graphene Nanosheets-350	3.01	16.358	0.742	44.34	5.38
Graphene Nanosheets-400	1.20	16.988	0.747	53.62	6.81
Graphene Nanosheets-450	16.40	14.525	0.67	45.74	4.46
Platinum	0.76	18.507	0.714	57.51	7.59

A higher V_oc_ is observed for the graphene sheets at 400 °C, which would account for a higher efficiency; however, it is low due to a decrease in the Jsc and FF; hence, platinum has a better efficiency.

In another study by Ibarra-Barreno et al., [131] they synthesized graphene CEs and obtained an efficiency of 6% whch is comparable to that of platinum (8%). his observed efficiency is attributed to the low short circuit voltage (11.8 mA/cm^2^) due to a high transfer resistance.

Furthermore a group by Kamesh et al., [132] designed graphene CEs which had an efficiency of 6.93% tht was comparable to that of platinum (7.35%). his was observed to increase due to the large surface area of graphene which improves the short circuit voltage (18.40 mA/cm^2^).

## Challenges and future prospects

Carbon nanotube and graphene counter electrodes have found wide application in DSSCs; however, further improvement in overall efficiency and performance has yet to be achieved. Several challenges are faced during the working operation of the counter electrodes. Future Prospects should also be addressed to enhance the performance and efficiency of the DSSCs.

### Challenges

1. Despite their high conductivity, CNTs and graphene can exhibit high charge transfer resistance at the interface with the electrolyte, leading to reduced cell efficiency. It is often caused by a passivating layer on the surface of these materials, hindering electron transfer.
2. CNTs and graphene are prone to aggregation, which can limit their effective surface area and reduce their catalytic activity. Ensuring proper dispersion of these materials in the CE layer is crucial for optimal performance.
3. Scaling up the production of these materials for large-scale DSSC manufacturing remains a challenge.
4. The long-term stability of CNT and graphene-based CEs in DSSCs can be affected by electrolyte degradation, corrosion, and mechanical stress. Ensuring the durability of these materials is essential for practical applications.

### Future prospects

1. Developing effective methods for dispersing CNTs and graphene in the CE layer and functionalizing their surfaces with catalytic groups such as hydroxyl, amino, and nitro can enhance charge transfer and reduce aggregation.
2. Combining CNTs and graphene with other materials, such as metal nanoparticles or conductive polymers, can create hybrid structures with synergistic properties, addressing the limitations of each material.
3. Research and development efforts are focused on reducing the production costs of CNTs and graphene through advancements in synthesis techniques, such as bottom-up approaches and large-scale manufacturing processes.
4. Exploring strategies to improve the durability of CNT and graphene-based CEs, such as protective coatings or using more stable electrolytes, is essential for long-term operation in DSSCs.
5. Beyond traditional DSSCs, CNT and graphene-based CEs have potential applications in other energy storage and conversion devices, such as batteries, fuel cells, and supercapacitors.

## Conclusions

The counter electrode collects the electrons from the external and brings them back to the cell by reducing the redox couple to create a continuous cycle. It is observed that the efficiency is influenced by the charge transfer resistance; that is, a low R_ct_ increases the efficiency while a high R_ct_ decreases the efficiency. Platinum is the commonly used electrode in DSSCs due to its high conversion efficiencies; however, due to its toxic nature, alternative materials are being applied. Carbon-based materials, that is, carbon nanotubes and graphene, have found wide application due to their abundance, low fabrication costs, and ease of modification. Carbon nanotubes, primarily single-walled carbon and double-walled, have good mechanical strength, electrical stability, and conductivity and hence have shown efficiencies of about 7.79%. This can be attributed to the thickness of the materials, which affects the morphology; thus, the electrical conductivity as the electrolyte quickly penetrates the surface, facilitating the regeneration of the redox mediator. Graphene has high transparency, large specific surface area, excellent thermal conductivity, and good carrier mobility properties, reducing charge transfer resistance. Furthermore, the structural defects and oxygen-containing sites improve the catalytic behaviour of graphene counter electrodes. Graphene can be synthesized in different physical forms, such as nanosheets and nanoplatelets; thus, its efficiency can be varied to suit the designed DSSCs. The carbon-based materials are cheap and readily available; hence, they have found wide applications as counter electrodes in different electronic and photoelectronic devices. In brief, this review confirms that carbon nanotubes and graphene are ideal candidates to replace platinum as counter electrodes for DSSCs in future electronic applications.

## Data Availability

No datasets were generated or analysed during the current study.
